# Data-driven FDG-PET subtypes of Alzheimer’s disease-related neurodegeneration

**DOI:** 10.1186/s13195-021-00785-9

**Published:** 2021-02-19

**Authors:** Fedor Levin, Daniel Ferreira, Catharina Lange, Martin Dyrba, Eric Westman, Ralph Buchert, Stefan J. Teipel, Michel J. Grothe, for the Alzheimer’s Disease Neuroimaging Initiative

**Affiliations:** 1 German Center for Neurodegenerative Diseases (DZNE), Rostock/Greifswald, Rostock, Germany; 2 Division of Clinical Geriatrics, Department of Neurobiology, Care Sciences and Society, Center for Alzheimer Research, Karolinska Institutet, Stockholm, Sweden; 3 Department of Nuclear Medicine, Charité – Universitätsmedizin Berlin, Corporate Member of Freie Universität Berlin, Humboldt-Universität zu Berlin and Berlin Institute of Health, Berlin, Germany; 4 German Center for Neurodegenerative Diseases (DZNE), Dresden, Germany; 5 Department of Neuroimaging, Centre for Neuroimaging Sciences, Institute of Psychiatry, Psychology and Neuroscience, King’s College London, London, UK; 6 Department of Diagnostic and Interventional Radiology and Nuclear Medicine, University Medical Center Hamburg-Eppendorf, Hamburg, Germany; 7 Department of Psychosomatic Medicine, University of Rostock, Rostock, Germany; 8 Unidad de Trastornos del Movimiento, Servicio de Neurología y Neurofisiología Clínica, Instituto de Biomedicina de Sevilla, Hospital Universitario Virgen del Rocío/CSIC/Universidad de Sevilla, Avda. Manuel Siurot, s/n, 41013 Sevilla, Spain

**Keywords:** Alzheimer’s disease, Subtypes, Mild cognitive impairment, Prodromal, FDG-PET, Hypometabolism

## Abstract

**Background:**

Previous research has described distinct subtypes of Alzheimer’s disease (AD) based on the differences in regional patterns of brain atrophy on MRI. We conducted a data-driven exploration of distinct AD neurodegeneration subtypes using FDG-PET as a sensitive molecular imaging marker of neurodegenerative processes.

**Methods:**

Hierarchical clustering of voxel-wise FDG-PET data from 177 amyloid-positive patients with AD dementia enrolled in the Alzheimer’s Disease Neuroimaging Initiative (ADNI) was used to identify distinct hypometabolic subtypes of AD, which were then further characterized with respect to clinical and biomarker characteristics. We then classified FDG-PET scans of 217 amyloid-positive patients with mild cognitive impairment (“prodromal AD”) according to the identified subtypes and studied their domain-specific cognitive trajectories and progression to dementia over a follow-up interval of up to 72 months.

**Results:**

Three main hypometabolic subtypes were identified: (i) “typical” (48.6%), showing a classic posterior temporo-parietal hypometabolic pattern; (ii) “limbic-predominant” (44.6%), characterized by old age and a memory-predominant cognitive profile; and (iii) a relatively rare “cortical-predominant” subtype (6.8%) characterized by younger age and more severe executive dysfunction. Subtypes classified in the prodromal AD sample demonstrated similar subtype characteristics as in the AD dementia sample and further showed differential courses of cognitive decline.

**Conclusions:**

These findings complement recent research efforts on MRI-based identification of distinct AD atrophy subtypes and may provide a potentially more sensitive molecular imaging tool for early detection and characterization of AD-related neurodegeneration variants at prodromal disease stages.

**Supplementary Information:**

The online version contains supplementary material available at 10.1186/s13195-021-00785-9.

## Introduction

Previous research has demonstrated heterogeneity in Alzheimer’s disease (AD) which is linked to distinct neuropathological subtypes of AD characterized by limbic-predominant, hippocampal sparing, or rather balanced (“typical”) spatial distributions of neurofibrillary tangle pathology [1]. Analysis of ante-mortem structural MRI data demonstrated that neuropathologically defined AD subtypes also show characteristic in vivo patterns of regional brain atrophy [2]. Recent research has used clustering methods on structural MRI data to identify similar regional atrophy subtypes in AD in a data-driven manner [3]. Interestingly, these atrophy subtypes could already be detected in patients with prodromal AD (i.e. amyloid-beta [Aβ]-positive patients with mild cognitive impairment [MCI]), who showed similar biomarker characteristics as the subtypes identified in patients with AD dementia and were associated with differential clinical trajectories [4, 5].

In addition to volumetric information from structural MRI, hypometabolism in FDG-PET is a well-established imaging marker of neurodegeneration as recognized by the recently revised research criteria for AD [6]. The use of FDG-PET in dementia and neurodegenerative cognitive impairment was also recently supported by consensus recommendations from a panel of experts from the European Association of Nuclear Medicine and the European Academy of Neurology [7]. FDG-PET may indicate a decrease in cerebral glucose metabolism that occurs prior to the macroscopic atrophy detectable with MRI, rendering the technique potentially more sensitive to early neurodegenerative processes [8–10]. While previous hypothesis-driven studies have also reported differential hypometabolic FDG-PET patterns amongst AD dementia patients [11–13], to our knowledge, FDG-PET has not yet been used for identifying neurodegeneration subtypes of AD in a data-driven manner. Given that detection of an “AD-typical” hypometabolism pattern by visual inspection of individual patients’ FDG-PET scans is commonly used for differential dementia diagnosis in research and clinical settings [7, 12, 14–16], a systematic evaluation of whether and how specific AD neurodegeneration subtypes are reflected in FDG-PET data can have important diagnostic implications.

In the current study, we investigated hypometabolic subtypes in patients with AD by applying an established hierarchical clustering approach to a large dataset of FDG-PET scans from patients with biomarker-confirmed AD enrolled in the Alzheimer’s Disease Neuroimaging Initiative (ADNI). The identified subtypes were further characterized with respect to detailed clinical and biomarker profiles and also used for subtype stratification of an independent ADNI sample of patients with prodromal AD (Aβ-positive patients with MCI) who were clinically followed for up to 72 months.

## Methods

### Participants

We included data from 179 cognitively normal (CN) participants (58 Aβ-positive), 177 Aβ-positive patients with a clinical diagnosis of AD dementia, and 217 Aβ-positive patients with MCI (i.e. prodromal AD) from the ADNI-1, ADNI-GO/2 and ADNI-3 cohorts (adni.loni.usc.edu). The detailed inclusion criteria for the different diagnostic categories have been described in detail before [17] and are available on the ADNI website (http://adni.loni.usc.edu/methods/documents/). Evidence of Aβ pathology was based on AV45-PET or, in case this measure was not available, on CSF Aβ levels (see below for details). This was only the case for 48 AD dementia patients (i.e. 12% of the total patient sample). The ADNI is a longitudinal multicentre study aimed at investigating whether neuroimaging methods such as MRI and PET, together with genetic, clinical and neuropsychological measures, can be used to characterize the progression of MCI and AD. The ADNI was launched in 2003 as a public-private partnership, led by principal investigator Michael W. Weiner, MD.

### Neuropsychological test scores

Cognitive performance in the ADNI is assessed using neuropsychological test batteries covering various cognitive domains. We used previously established composite cognitive scores for memory (ADNI-MEM), executive function (ADNI-EF), visuospatial function (ADNI-VS) and language (ADNI-Lan) [18–20]. Mini-Mental State Examination (MMSE) scores were used for characterizing global cognitive impairment. We also calculated the difference between the ADNI-MEM and the ADNI-EF composite scores (ADNI-DIFF) to characterize differential decline in these two domains. Positive values in this variable thus represent a more pronounced executive impairment compared to the memory deficit, and vice versa for negative values.

### Longitudinal analysis

We analysed longitudinal changes in cognitive functions of the prodromal AD subtypes for participants with available follow-up data (*n* = 200). We used longitudinal measures of ADNI-MEM, ADNI-EF, ADNI-VS and ADNI-Lan. Additionally, longitudinal Clinical Dementia Rating (CDR) scores were used as a criterion indicating a progression from prodromal (CDR = 0.5) to clinically manifest AD dementia (CDR ≥ 1). The mean follow-up period was 44 months (range 12–72 months), and 72.5% of participants had at least 36 months of follow-up available.

### Biomarkers

Measures of cortex-to-whole cerebellum AV45 standard uptake value ratios (SUVR) have been calculated by the ADNI PET core (Jagust Lab, UC Berkeley) and were downloaded from the ADNI server. We selected Aβ-positive patients with AD or MCI if their AV45 SUVR values were greater than or equal to the recommended threshold of 1.11 [21].

ADNI CSF values in the current study were derived from electrochemiluminescence immunoassays for Aβ [1–42], tau phosphorylated at threonine 181 (p-tau) and total tau (t-tau) on an automated Elecsys cobas e 601 instrument.^1^ We included participants who had CSF Aβ values lower than the threshold of 880 pg/ml proposed by Hansson et al. [22].

Selecting patients based on biomarker evidence of amyloidosis follows the design of previous MRI-based subtyping studies of differential neurodegeneration patterns in AD [4, 5]. However, given that the 2018 NIA-AA revised research criteria now reserve the term “Alzheimer’s disease” for combined evidence of amyloid and tau positivity [6], we also categorized patients with regard to the full A/T/(N) classification using CSF p-tau levels above 19.2 pg/ml for denoting tau biomarker (T) positivity and CSF t-tau levels above 242 pg/ml for denoting neurodegeneration biomarker (N) positivity [23]. Full CSF biomarker data was available for 93% of our study sample (Supplementary Table 1).

*APOE* genotype was determined using DNA extracted from a 3-ml aliquot of EDTA blood samples by Cogenics [24]. Genotype information was coded in a binary *APOE* ε4 variable indicating the presence of at least one *APOE* ε4 allele.

Structural MRI images were used to derive hippocampal and cortical volume measures. MRI images in ADNI are acquired at multiple sites using scanner-specific T1-weighted sagittal 3D MPRAGE sequences and undergo standardized image pre-processing steps to improve uniformity across the scanners (http://adni.loni.usc.edu/methods/documents/). We extracted regional grey matter volumes from these scans using a previously described automated volumetry approach implemented in statistical parametric mapping software (SPM8, Wellcome Trust Center for Neuroimaging) and the VBM8-toolbox [25, 26]. Briefly, this involves automated tissue class segmentation and high-dimensional spatial normalization to an ageing/AD-specific reference template. Spatially normalized grey matter (GM) maps were visually inspected for segmentation and normalization accuracy, and voxel values were modulated for volumetric changes introduced by the high-dimensional normalization, so that the total GM volume present before warping was preserved. Hippocampal (HV) and regional cortical grey matter volumes were extracted from these scans using regions-of-interest defined in the Harvard-Oxford anatomical atlas [27]. Individual volumes were divided by the total intracranial volume (TIV), calculated as the sum of total volumes of all tissue segments. In analogy to previous MRI-based subtyping studies [2, 28], cortical grey matter volumes were extracted from selected frontal, temporal and parietal association areas (see Supplementary Table 2), summed into a measure of bilateral cortical total volume (CTV), and further used to calculate the hippocampal to cortical volume ratio (HV:CTV).

Finally, we included white matter hyperintensity (WMH) volume as a measure of small vessel vascular disease burden. These values have been calculated by the ADNI MRI core and were downloaded from the ADNI server. In ADNI-1 data, WMH values were obtained via analysis of the proton density (PD) and T1 and T2 MRIs [29]. In ADNI-GO/2, a fluid-attenuated inversion recovery (FLAIR) MRI sequence was used to calculate WMH volumes [30]. In the present study, we pooled available WMH measures [4] and controlled statistical analyses of this variable for different segmentation methods using a dummy-coded confound variable.

### FDG-PET data acquisition and preprocessing

FDG-PET data were retrieved in a pre-processed form from the ADNI server. FDG-PET images were obtained on multiple scanners with protocols specific to platforms. Dynamic 3D scans of six 5-min frames were acquired 30–60 min after injections of 185 MBq of ^18^F-FDG. All original ADNI FDG-PET scans underwent standardized image pre-processing steps to improve uniformity across the scanners. Detailed information on FDG-PET acquisition and pre-processing is available on the ADNI website (http://adni.loni.usc.edu/methods/documents/). For the present study, FDG-PET images were further spatially normalized to a customized FDG-PET standard space template and smoothed with a Gaussian smoothing kernel of 8 mm full-width at half maximum (FWHM) using SPM8 [31].

### Hierarchical clustering

The patients with AD dementia were classified into hypometabolic subtypes using agglomerative hierarchical clustering of voxel-wise FDG-PET data with Ward’s linkage as implemented in the MATLAB software [32, 33]. Individual FDG-PET profiles were scaled to their global mean prior to clustering analysis so that clustering relied on the differences in regional hypometabolic patterns rather than on potential differences in global hypometabolism severity across patients [32]. Global intensity scaling is a commonly used approach in neuroimaging-based subtyping studies to control clustering analyses for individual differences in disease severity (see [3] for a recent review of this literature). Accordingly, similar global scaling methods were also used in previous FDG-PET studies that employed hierarchical clustering approaches for data-driven characterizations of FDG-PET subtypes in other neurodegenerative dementias [32, 34].

In the clustering procedure, the algorithm progressively combines closest voxel-wise FDG-PET profiles of the participants into larger clusters, as well as most similar clusters with each other. The output of the algorithm is a hierarchical dendrogram in which the level of branching indicates the degree of dissimilarity between the clusters. The optimal number of separable clusters in the data was evaluated using standard performance measures for clustering solutions including the Davies-Bouldin criterion [35] and the silhouette criterion [36].

To visualize the patterns of hypometabolism in the identified subtypes, we conducted voxel-wise two-sample *t* tests between FDG-PET images from each of the subtypes and the CN group, using age, gender and years of education as covariates. In contrast to the global signal scaling used in the clustering procedure, images were scaled to the average signal in a pons reference region for this analysis as the standard method to assess hypometabolism in comparison with a healthy control group [31]. Global signal scaling is typically not recommended in this context, because it accounts for global disease-related differences in glucose utilization that typically exist between dementia patients and healthy controls and thus lowers the sensitivity to detect regional hypometabolism [37, 38]. An explicit grey matter mask was applied to the images, and obtained *t* values were converted into Cohen’s *d* effect size values. Analogous voxel-wise analyses were also conducted to directly compare the different subtypes identified in the AD group.

### Classification of patients with prodromal AD

We classified FDG-PET scans of patients with prodromal AD according to the identified AD subtypes using a fully automated classification procedure. For that, we first screened patients for evidence of regional hypometabolism by assessing whether at least one of the 48 bilateral cortical areas defined in the Harvard-Oxford atlas had an FDG-PET signal (scaled to pons) of at least one standard deviation below the mean of the control group. Participants with no such regions were classified into a “no hypometabolism” subtype [12, 14]. The remaining patients with prodromal AD were classified into one of the subtypes identified in the AD dementia group based on the smallest Euclidean distance between the individual patient’s voxel-wise FDG-PET profile (scaled to global values) and the mean FDG-PET profile of each of the AD dementia subtypes [4]. Global scaling was used for this classification procedure in order to match the individual FDG patterns of the MCI patients to the regional patterns defined by the AD subtypes independent of global hypometabolism severity. Visualization of the hypometabolism patterns of the classified subtypes as compared to the CN group employed identical voxel-wise two-sample *t* tests of pons-scaled FDG-PET images as described above.

### Statistical analysis

Statistical analyses were conducted using RStudio and R version 3.5.2 with a statistical significance threshold of *P* <  0.05 (two-tailed). Chi-squared tests with post hoc pairwise proportion tests were used to compare gender compositions of subtypes and frequencies of the *APOE* ε4 genotype. For this and other post hoc tests comparing the subtypes, we used the false discovery rate (FDR) correction [39] as implemented in R. Age and years of education were compared across subtypes using ANOVA. Differences in cognitive measures and biomarkers across the subtypes were tested with ANCOVA using age, gender and education as covariates.

For patients with prodromal AD with available clinical follow-up data, we also conducted Cox proportional hazards regression analyses for analysing differential risks of progression to dementia across FDG-PET defined subtypes. Progression to dementia was operationalized as a change in CDR score from 0.5 to ≥ 1 [40]. Models included age, gender and education as covariates. Participants were censored if they did not progress to dementia before the last available follow-up CDR score.

In addition, linear mixed effects regression models were used to assess the differences in domain-specific longitudinal changes in memory, executive function, visuospatial function or language function [40, 41]. Models included the time of follow-up measured in months from baseline, a factor variable indicating subtype and an interaction term for the time by subtype as independent variables. The estimates for interactions between subtype and time indicated whether subtypes had differential cognitive trajectories over time. Age, gender and education were included as covariates. Regression models included random intercepts and random slopes for participants; *t* tests used Satterthwaite approximations for degrees of freedom.

## Results

### Identification of hypometabolic subtypes in patients with AD dementia

**Fig. 1:**
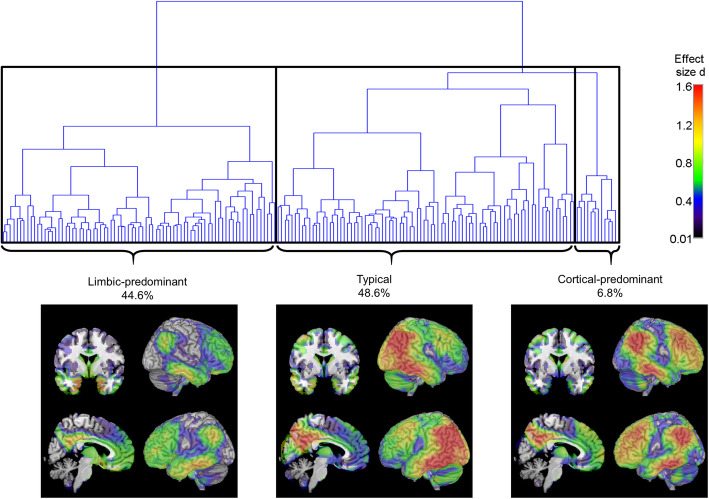
Hierarchical clustering dendrogram and hypometabolic FDG-PET patterns of identified AD subtypes. Dendrogram resulting from Ward’s hierarchical clustering analysis of individual FDG-PET profiles of patients with AD dementia. Brain plots show voxel-wise hypometabolic patterns of the three identified AD subtypes as revealed by statistical comparison to the healthy control group. FDG-PET scans were scaled to the average pons signal prior to the group comparisons, and age, gender, and years of education were used as covariates. Statistical parametric maps of the group differences were converted into Cohen’s *d* effect size maps to allow for a better comparison of the patterns across the unevenly sized AD subgroups. Subtype patterns at higher clustering solutions are shown in Supplementary Figure 2

We used objective criteria for evaluating optimal clustering solutions to select the level of cutoff for the hierarchical clustering dendrogram supported by the data. The Davies-Bouldin criterion favoured solutions with three or five clusters, whereas the silhouette criterion favoured three clusters (see Supplementary Figure 1). Therefore, we chose the clustering solution with three clusters (see results for solutions with higher cluster numbers in the Supplementary Figure 2). Cluster-1 included 44.6% of the patients and showed a predominantly limbic hypometabolic profile with most pronounced hypometabolism in the medial temporal lobe, which extended to the posterior cingulate cortex, lateral temporo-parietal areas and also large areas of the ventromedial and lateral frontal lobe (Fig. 1, henceforth referred to as the “limbic-predominant” subtype). Cluster-2 included 48.6% of the patients and corresponded to a more AD-typical pattern of marked posterior temporo-parietal hypometabolism with additional, albeit less pronounced, involvement of the medial temporal lobe (henceforth referred to as the “typical” subtype). Cluster-3 included 6.8% of the patients and showed a pattern of temporo-parietal cortical hypometabolism similar to that of cluster-2, but with more extensive involvement of the frontal lobe and largely spared metabolism in the medial temporal lobe (henceforth referred to as the “cortical-predominant” subtype). Direct comparisons of regional hypometabolism between subtypes are shown in Supplementary Figure 3.

### Clinical and biological characterization of the hypometabolic AD dementia subtypes

Demographic, clinical and biomarker characteristics of the three AD subtypes are listed in Table 1. The “limbic-predominant” hypometabolic subtype was the oldest with an average age of 75.4 years and the “cortical-predominant” subtype the youngest with an average age of 68.0 years. The “limbic-predominant” subtype generally showed the least pronounced impairments in neuropsychological testing and was characterized by a memory-predominant cognitive profile, whereas in the “typical” and especially the “cortical-predominant” subtype, the impairment in executive functions exceeded the mnestic deficit. Reflecting the subtype-defining hypometabolic patterns, hippocampal volume was lowest in the “limbic-predominant” subtype and highest in the “cortical-predominant” subtype, whereas cortical volumes showed the opposite behaviour. The resulting HV:CTV ratio was significantly higher for the “cortical-predominant” subtype in comparison with the two other subtypes. The “cortical-predominant” subtype also had a strikingly lower percentage of *APOE* ε4 carriers compared to the other two subtypes, although this difference did not reach statistical significance. No differences were observed between the subtypes with regard to gender distribution, years of education, molecular biomarkers of Aβ and tau pathology burden, or WMH volume (Table 1).

**Table 1:** Demographic, clinical and biomarker characteristics of AD dementia subtypes at baseline

	CN group	AD group, limbic-predominant (S1)	AD group, typical (S2)	AD group, cortical-predominant (S3)	*P* value, global comparison (S1, S2 and S3)	Pair-wise comparisons		
						S1 vs S2	S1 vs S3	S2 vs S3
**Demographics**								
*n* (%)	179	79 (44.6%)	86 (48.6%)	12 (6.8%)				
Age, years	73.8 (6.5)	75.4 (6.9)	73.2 (5.7)	68.0 (7.7)	0.007	0.149	0.015	0.088
Sex, female (%)	50%	49%	38%	50%	0.332			
Education, years	16.6 (2.5)	15.4 (3.1)	15.5 (2.6)	16.3 (2.6)	0.544			
**Cognition**								
MMSE	29.1 (1.2)	23.4 (1.9)	23.2 (2.2)	22.0 (2.2)	0.037	0.798	0.165	0.200
ADNI-MEM	1.04 (0.62)	− 0.85 (0.55)	− 0.90 (0.49)	− 1.31 (0.44)	0.012	1	0.025	0.028
ADNI-EF	0.92 (0.83)	− 0.65 (0.86)	− 1.11 (0.89)	− 1.73 (0.81)	< 0.001	0.002	< 0.001	0.043
ADNI-DIFF	0.13 (0.72)	− 0.21 (0.69)	0.21 (0.85)	0.41 (0.74)	< 0.001	0.004	0.029	0.723
ADNI-VS	0.23 (0.59)	− 0.42 (0.8)	− 0.67 (1.01)	− 1.15 (1.08)	0.085			
ADNI-Lan	0.89 (0.71)	− 0.63 (0.95)	− 0.83 (0.88)	− 1.11 (0.78)	0.03	0.406	0.406	0.588
**Biomarkers**								
APOE ε4 (%)	28%	81%	79%	58%	0.222			
AV45-PET SUVR	1.11 (0.18)	1.43 (0.14)	1.47 (0.17)	1.4 (0.17)	0.145			
CSF Aβ, pg/ml	1392 (663)	598 (163)	585 (225)	629 (169)	0.686			
CSF t-tau, pg/ml	236 (92)	374 (143)	374 (154)	402 (124)	0.978			
CSF p-tau, pg/ml	22 (9)	38 (16)	37 (16)	39 (14)	0.881			
HV	4.97 (0.38)	4.05 (0.51)	4.1 (0.38)	4.46 (0.46)	0.09			
CTV	87.39 (6.28)	75.84 (6.95)	73.99 (6.23)	70.04 (5.88)	0.006	0.136	0.026	0.136
HV:CTV ratio	57.11 (5.29)	53.67 (7.57)	55.74 (6.61)	64.29 (10.08)	< 0.001	0.136	< 0.001	< 0.001
WMH	6.1 (10.4)	8.0 (10.0)	5.8 (7.9)	8.1 (12.7)	0.262			

Values for variables are presented as percentages (for gender and APOE ε4 genotype) or means with standard deviation in parentheses. Missing values are excluded (for numbers of missing values per subtype see Supplementary Table 8). In case of significant main effects, subtypes were compared with the post hoc pairwise *t* tests with FDR correction. Please note that composite cognitive scores have arbitrary units on scales centred on the original test samples used to develop these scores (including patients and healthy controls) [18–20]

*S1* limbic-predominant subtype, *S2* typical subtype, *S3* cortical-predominant subtype, *HV* hippocampal grey matter volume scaled to total intracranial volume, *CTV* cortical composite grey matter volume scaled to total intracranial volume

### Stratification of prodromal AD patients by hypometabolic subtypes

**Fig. 2:**
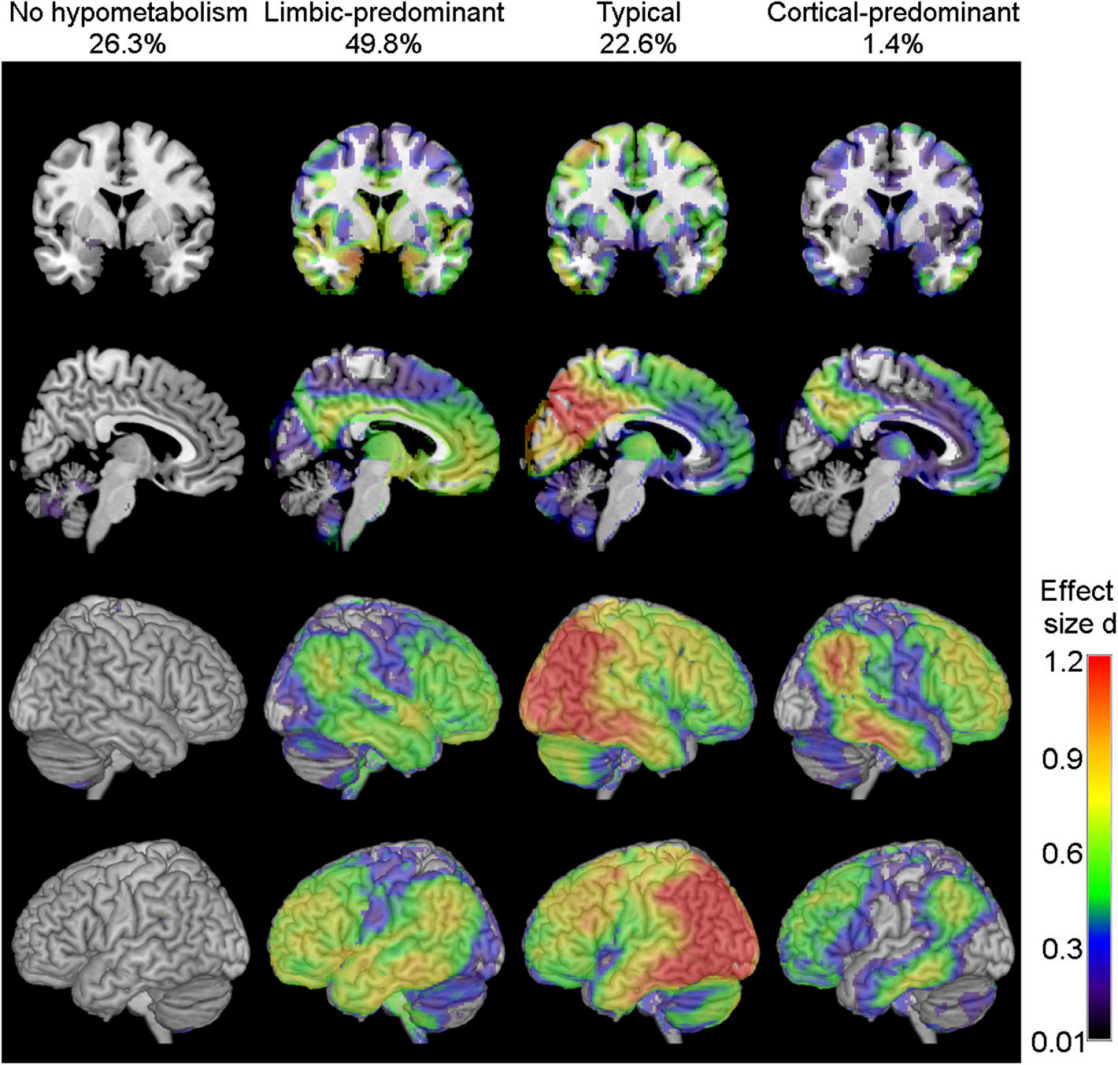
Hypometabolic FDG-PET patterns of subtypes of patients with prodromal AD. Voxel-wise hypometabolic patterns of the four prodromal AD subtypes as compared to the healthy control group. FDG-PET scans were scaled to the average pons signal prior to analysis, and age, gender, and years of education were used as covariates. Statistical parametric maps of the group differences were converted into Cohen’s *d* effect size maps to allow for a better comparison of the patterns across the unevenly sized subgroups. Please note that the effect size scale differs from the scale used in Fig. 1 as it has been adapted to optimally display the differential patterns that define the subtypes at the prodromal disease stage. Supplementary Figure 4 provides an illustration of the hypometabolic patterns in the prodromal group when using the same effect size scale as in the AD dementia group

About a quarter (26.3%) of the prodromal AD cohort did not show any evidence of regional hypometabolism and was thus classified as “no hypometabolism” subtype. Amongst the other patients with prodromal AD, 49.8% were classified into the “limbic-predominant” subtype, 22.6% into the “typical” subtype and only three participants (1.4%) were classified into the “cortical-predominant” subtype, which was thus omitted from further analyses. Hypometabolic patterns of the classified subtypes in the prodromal AD cohort showed a strong spatial resemblance with the subtype-defining patterns in the AD dementia cohort (Fig. 2), thus corroborating the validity of the automated classification procedure. Demographic, clinical and biomarker characteristics of the subtypes are summarized in Table 2. The “limbic-predominant” subtype was again characterized by a significantly higher age compared to the other subtypes. The “no hypometabolism” subtype was the youngest and also had a higher proportion of females. Despite no pairwise differences in MMSE scores, the “no hypometabolism” subtype also showed significantly better memory and executive function performance than the “typical” and the “limbic-predominant” subtypes. Although we selected only Aβ-positive patients, the “no hypometabolism” subtype showed a significantly lower Aβ burden on both PET and CSF measures. Nevertheless, patients in the “no hypometabolism” subtype showed a comparably high proportion of concomitant tau biomarker positivity as the other subtypes in the prodromal AD group (86–90%; Supplementary Table 1). In accordance with the FDG-PET characteristics, the “no hypometabolism” subtype had significantly higher hippocampal and cortical volumes compared to the “limbic-predominant” and the “typical” subtypes.

**Table 2:** Demographic, clinical and biomarker characteristics of prodromal AD subtypes at baseline

	Prodromal AD group, no hypometabolism (S0)	Prodromal AD group, limbic-predominant (S1)	Prodromal AD group, typical (S2)	*P* value, global comparison (S0, S1 and S2)	Pair-wise comparisons		
					S0 vs S1	S0 vs S2	S1 vs S2
**Demographics**							
*n* (%)	57 (26.3%)	108 (49.8%)	49 (22.6%)				
Age, years	68.4 (6.6)	76.1 (5.7)	71.7 (6.2)	< 0.001	< 0.001	0.009	< 0.001
Sex, female (%)	60%	35%	41%	0.01	0.024	0.226	1
Education, years	16.2 (2.8)	15.7 (3.0)	16.4 (2.6)	0.344			
**Cognition**							
MMSE	28.2 (1.8)	27.6 (1.8)	27.3 (1.8)	0.053			
ADNI-MEM	0.57 (0.63)	0.05 (0.61)	− 0.06 (0.65)	< 0.001	< 0.001	< 0.001	0.602
ADNI-EF	0.76 (0.91)	0.00 (0.77)	0.15 (1.01)	0.018	< 0.001	0.001	0.542
ADNI-DIFF	− 0.19 (0.83)	0.05 (0.78)	− 0.21 (0.77)	0.616			
ADNI-VS	0.08 (0.65)	− 0.06 (0.76)	− 0.21 (0.67)	0.135			
ADNI-Lan	0.6 (0.7)	− 0.05 (0.74)	0.29 (0.79)	0.007	< 0.001	0.062	0.025
**Biomarkers**							
APOE ε4 (%)	68%	62%	77%	0.163			
AV45-PET SUVR	1.31 (0.17)	1.39 (0.17)	1.43 (0.15)	0.004	0.015	0.002	0.238
CSF Aβ, pg/ml	921 (437)	736 (237)	672 (214)	0.002	< 0.001	< 0.001	0.417
CSF t-tau, pg/ml	315 (134)	337 (137)	357 (144)	0.123			
CSF p-tau, pg/ml	31 (15)	34 (16)	37 (16)	0.096			
HV	4.86 (0.45)	4.53 (0.51)	4.57 (0.41)	0.009	< 0.001	0.005	1
CTV	89.87 (5.45)	83.19 (6.48)	82.93 (5.35)	< 0.001	< 0.001	< 0.001	1
HV:CTV ratio	54.26 (6.07)	54.57 (6.27)	55.24 (5.56)	0.344			
WMH	4.9 (5.0)	11.3 (12.8)	6.8 (6.5)	0.172			

Cortical-predominant subtype of prodromal AD group (*n* = 3) not included. Values for variables are presented as percentages (for gender and APOE ε4 genotype) or means with standard deviation in parentheses. Missing values are excluded (for numbers of missing values per subtype, see Supplementary Table 8). In case of significant main effects, subtypes were compared with the post hoc pairwise *t* tests with FDR correction. Please note that composite cognitive scores have arbitrary units on scales centred on the original test samples used to develop these scores (including patients and healthy controls) [18–20]

*S0* no hypometabolism subtype, *S1* limbic-predominant subtype, *S2* typical subtype, *HV* hippocampal grey matter volume scaled to total intracranial volume, *CTV* cortical composite grey matter volume scaled to total intracranial volume

**Fig. 3:**
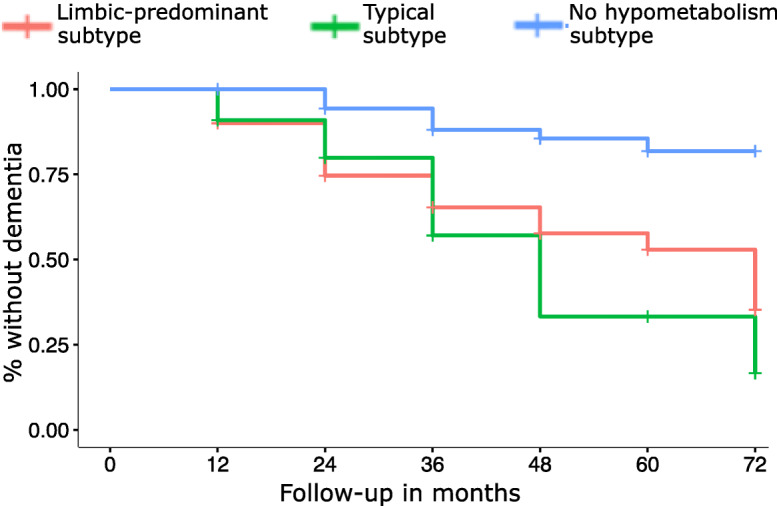
Kaplan-Meier curves of the time to progression to dementia across subtypes in the prodromal AD group. Kaplan-Meier survival curves indicate proportions of participants within the three prodromal AD subtypes progressing to dementia, operationalized as a change in CDR score from 0.5 to ≥ 1. Patients who did not progress to dementia within the observation period or did not have follow-up CDR scores were censored

**Fig. 4:**
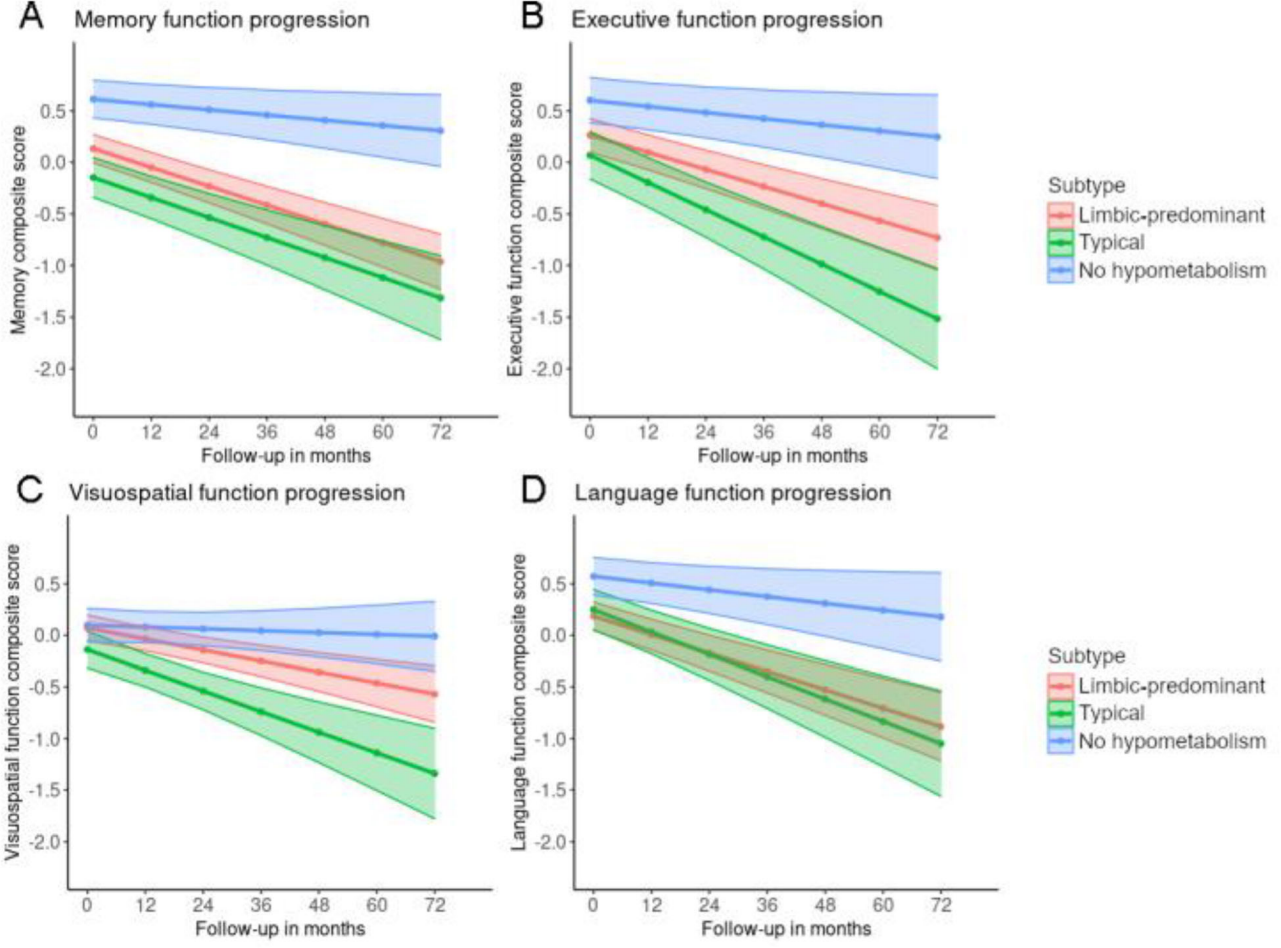
Longitudinal cognitive trajectories of subtypes of patients with prodromal AD. Predicted values of domain-specific cognitive scores were obtained from mixed effects regression models which included age, gender, and years of education as covariates, as well as random intercepts and slopes for participants to account for multiple measurements. **a** Memory function progression. **b** Executive function progression. **c** Visuospatial function progression. **d** Language function progression. Ribbons around the regression lines represent 95% confidence intervals for the fitted values

Analysis of longitudinal clinical data showed that the “no hypometabolism” subtype was at the lowest risk of progression to dementia (vs “limbic-predominant”: HR, 4.82, *P* < 0.001; vs “typical”: HR, 5.99, *P* < 0.001; Fig. 3) and also showed a significantly slower decline in all four cognitive domains compared to the “typical” and the “limbic-predominant” subtypes (Fig. 4, see Supplementary Tables 3 and 4 for full model stats). Interestingly, the “typical” and “limbic-predominant” subtypes showed a similar decrease in memory function over time, but the “typical” subtype showed a significantly faster decline in executive function (*t* = − 2.25, *P* = 0.026) and a trend towards faster decline in visuospatial functions (*t* = − 1.931, *P* = 0.055; see Supplementary Table 5 for full model stats).

## Discussion

In the current study, we conducted the first data-driven characterization of systematic heterogeneity in individual-level FDG-PET patterns amongst AD dementia patients, and provide evidence for the existence of three distinct hypometabolic subtypes of AD. These subtypes include a “typical” subtype of posterior temporo-parietal hypometabolism, as well as distinct “limbic-predominant” and “cortical-predominant” subtypes that resemble previously described MRI-based atrophy subtypes of AD and show corresponding differences in their clinical profiles. By stratifying an independent sample of longitudinally followed prodromal AD patients according to hypometabolic subtype, we could further demonstrate that these subtypes can be detected at a prodromal disease stage and are characterized by differential courses of cognitive decline.

### Distinct hypometabolic subtypes amongst patients with AD dementia

The “typical” subtype included the largest portion of AD dementia cases and was characterized by a typical posterior temporo-parietal pattern of hypometabolism that is commonly linked to AD [16, 42]. The “limbic-predominant” subtype had most pronounced hypometabolism in the hippocampus and related medial temporal structures which showed similarities to the MRI-defined medial temporal-dominant atrophy subtypes [4, 33, 43, 44]. Despite these similarities, the “limbic-predominant” subtype in the current study notably differs from previously described medial temporal-dominant atrophy subtypes by showing a more extensive hypometabolic pattern covering widespread limbic areas beyond the medial temporal lobe and including the frontal cortex. A previous study examining FDG-PET patterns in MRI-defined atrophy subtypes also reported pronounced frontal hypometabolism in the medial temporal-dominant AD atrophy subtype [44]. This could potentially be attributed to differences in the specific pathologic substrate of hypometabolism on FDG-PET and grey matter reductions on MRI as neurodegeneration markers. Specifically, hypometabolism on FDG-PET has been reported to be sensitive to neurodegenerative dysfunction that does not regionally co-localize with neuronal loss as measured by grey matter atrophy on MRI, which may reflect early non-macroscopic neurodegenerative processes or functional changes caused by atrophy in remote but functionally interconnected brain areas [8, 17, 45–47].

Similarly to previous findings on the MRI-defined medial temporal-predominant atrophy subtype [4, 43], the “limbic-predominant” hypometabolic subtype in the current study was associated with older age and could possibly reflect the effects of comorbid age-related pathologies. For example, Zhang et al. [5] considered that the temporal factor described in their study could be linked to comorbid TDP-43 pathology. Indeed, the hypometabolic pattern of the “limbic-predominant” subtype identified in the present study shows a striking resemblance with a recently described FDG-PET pattern of pathologically confirmed patients with AD dementia with comorbid TDP-43 pathology and hippocampal sclerosis (HS) [48] (specifically, Fig. 3, page 1209 in that study). Corroborating this qualitative visual interpretation, in a complementary post-hoc analysis, we found that the “limbic-predominant” subtype had a significantly higher inferior-to-medial temporal FDG-PET ratio compared to both the “typical” and “cortical-predominant” subtypes (Supplementary Table 6). This ratio has been suggested to reflect the difference between the TDP-43/HS-related pattern and the AD-typical pattern within a simplified metric and has been proposed as an imaging biomarker for comorbid TDP-43/HS in AD [48, 49]. However, it is important to note that any possible involvement of comorbid pathologies in the observed hypometabolic subtypes remains entirely speculative in our in vivo neuroimaging study, and we did not observe any notable differences in the proportion of AD-specific A/T biomarker profiles in the limbic-predominant subtype compared to the other subtypes (Supplementary Table 1). Other studies suggested that the medial-temporal subtype could be additionally affected by small vessel disease [4, 50], which would coincide with the numerically highest WMH volume in the “limbic-predominant” subtype in our study. However, this difference did not reach statistical significance in our analysis. Additional neuropathologic examinations as well as mechanistic studies are needed to better understand the exact pathological substrates and neurobiological mechanisms that drive different neurodegeneration subtypes in AD.

The hypometabolic pattern of the “cortical-predominant” subtype was similar to that of the “typical” subtype, but with more extensive involvement of the frontal lobe and largely normal metabolism in the medial temporal lobe. This subtype showed particularly pronounced executive function impairment in addition to the memory deficit. Previously, studies by Collette et al. [11] and Mosconi et al. [12] have also described marked frontal hypometabolism in subsets of patients with AD. On the other side, Ossenkoppele et al. [51] described an autopsy/biomarker-confirmed dysexecutive AD variant, which shows markedly more pronounced impairment in executive function relative to the memory deficit and is characterized by early onset of AD and a relatively low *APOE* ε4 frequency. Similarly, the “cortical-predominant” subtype in the current study also showed the youngest age and lowest percentage of *APOE* ε4 carriers amongst AD subtypes. However, due to the low number of patients in this group, current findings on this subtype require further corroboration.

In accordance with the subtype-defining hypometabolic patterns, we observed a difference between subtypes in the HV:CTV ratio. Specifically, it was the highest for the “cortical-predominant” subtype, intermediate for the “typical” and numerically the lowest for the “limbic-predominant” subtype. The pattern of differences in HV:CTV ratio between FDG-PET subtypes in the current study is comparable to previous findings on AD subtypes based on neuropathological data or MRI-based atrophy patterns. The study by Whitwell et al. [2] examined AD subtypes based on neuropathological examination of distribution of neurofibrillary tangle counts. The ratio between hippocampal and cortical volumes measured on ante-mortem MRI allowed for the best discrimination between these subtypes. In their study, similarly to our results, the typical subtype showed a higher HV:CTV ratio than the limbic-predominant subtype, whereas the hippocampal sparing subtype had the highest value. Furthermore, in the study by Risacher et al. [28], three AD subtypes—hippocampal sparing, limbic predominant and typical AD—were defined based on the HV:CTV ratio. Across these subtypes, a higher HV:CTV ratio was also quantitatively associated with a more pronounced dysexecutive profile, similarly to the differences observed for the ADNI-EF and ADNI-DIFF variables between the subtypes in the current study. In a complementary analysis, we could also reproduce this association between HV:CTV ratio and cognitive profile on a continuous scale (Supplementary Table 7). Thus, across patients, the HV:CTV ratio correlated positively with the ADNI-DIFF variable in both the AD dementia and prodromal AD groups, indicating a more pronounced executive function over memory deficit for higher values of this ratio. Therefore, the HV:CTV ratio measured in the current subtypes provides a link between our findings on hypometabolism subtypes and previously characterized AD subtypes based on neuropathological data or MRI-based atrophy patterns. However, there were also differences between the observed hypometabolism subtypes and previously reported atrophy patterns on MRI, such as the aforementioned more extensive involvement of the temporal and frontal areas in the “limbic-predominant” hypometabolism subtype, which might be attributed to the different structural and functional substrates of the respective imaging methods.

### Stratification of prodromal AD patients according to hypometabolic subtype

Stratification of the prodromal AD cohort according to hypometabolic subtypes revealed a considerably sized subgroup of patients with no or only minimal hypometabolism. This “no hypometabolism” subtype also showed lower levels of Aβ biomarker burden, was less cognitively impaired and had a lower risk of progressing to dementia compared to the other subtypes. While this may indicate that this subtype may be enriched for patients with only incidental amyloidosis, the comparably high proportion of concomitant tau biomarker positivity in this group (Supplementary Table 1) would rather argue against this possibility.

Previous studies using visual classification of FDG-PET scans had also described subsets of patients with MCI without evidence of regional hypometabolism [12, 14]. In the study by Cerami et al. [14], 31% of participants with MCI showed normal brain metabolism, although the large majority of these also had a negative amyloid biomarker finding. However, MRI-based subtyping studies have also consistently identified subsets of patients with AD dementia with no or only minimal atrophy, and this subtype was particularly prevalent amongst patients with prodromal AD [4, 43, 52]. Interestingly, in our study, we observed a similar “minimal” hypometabolism subtype in the AD dementia group when using a higher clustering solution (see Supplementary Figure 2). However, since we established the best distinguishable AD subtypes using an objective hierarchical clustering cutoff as suggested by the Davies-Bouldin and the silhouette criteria, we did not further characterize this “minimal” subtype in our study. Nevertheless, our findings underline the importance of accounting for the considerably sized subgroup of patients with prodromal AD without evidence of regional hypometabolism when characterizing heterogeneity of hypometabolism patterns in this population.

Only three participants with prodromal AD were classified into the “cortical-predominant” subtype and could thus not be further analysed in our study. One potential explanation for this low prevalence could be that this hypometabolic subtype is characterized by more pronounced executive function deficits than memory deficits from its prodromal stage on, so that these patients would be underrepresented in an MCI cohort screened for memory deficits such as the ADNI cohort.

The “limbic-predominant” and “typical” subtypes classified in the prodromal AD sample demonstrated similar subtype characteristics as in the AD dementia sample. Thus, the “limbic-predominant” subtype also had older age, numerically higher WMH volume and the most severe degree of hippocampus atrophy. Hence, current results confirm previous findings that the heterogeneity evident in patients with AD dementia can also be observed at the prodromal stage of the disease [4, 5]. Interestingly, although the “limbic-predominant” and “typical” subtypes had a comparable risk of progressing to dementia, the “limbic-predominant” subtype showed a more memory-selective cognitive decline compared to the “typical” subtype, paralleling the cross-sectional subtype differences in cognitive profiles observed in the AD dementia cohort. This finding is notable, because these subsets of patients with prodromal AD did not show significant differences in the respective cognitive functions at baseline. This indicates that subtype classification of FDG-PET patterns may provide additional information for predicting future cognitive decline that is not contained in neuropsychological assessments.

Our current findings on subtype-specific trajectories of cognitive decline are largely consistent with previous findings on differences in dementia risk and domain-specific cognitive decline between atrophy subtypes based on MRI data. For example, the studies by Ten Kate et al. [4] and Dong et al. [5] both only found a significantly lower risk for progression to dementia in the no/minimal atrophy subtype, and while the other atrophy subtypes showed similar overall risk for progression to dementia, they differed significantly in the relative decline in specific cognitive domains.

A previous study by Morbelli et al. [53] used classical voxel-wise analyses of FDG-PET images in a group of prodromal AD patients to determine a “prognostic pattern” of regional brain hypometabolism that best correlated with time to conversion to dementia. Interestingly, this “prognostic pattern” was found to be considerably different from the AD-typical “diagnostic pattern” of hypometabolism as determined by contrasting AD patients with healthy controls. In our current study, we used a data-driven approach to demonstrate that this typical group-averaged “diagnostic pattern” can be decomposed into different regional subtypes corresponding to distinct subgroups of AD patients. It is likely that these different FDG-PET subtypes also correspond to different “prognostic patterns” that best predict time to dementia conversion in the respective subgroups of prodromal AD patients.

### Strengths and limitations

One conceptual strength of the current study is that we only included patients with biomarker evidence of Aβ pathology. Moreover, we used the identified hypometabolism subtypes in patients with AD dementia to classify an independent dataset of Aβ-positive patients with MCI and assess clinical and biomarker characteristics of these subtypes at a prodromal disease stage.

As with other unsupervised subtyping studies, a principal limitation of the current study is that the employed clustering methodology cannot naturally distinguish between subtypes and different disease stages. We aimed to mitigate the effect of differing disease stages by normalizing the individual FDG-PET profiles to their global signal before clustering, so that the cluster assignations were primarily driven by relative regional metabolic differences instead of global differences accompanying disease progression. Global signal scaling is a commonly used method to control neuroimaging clustering analyses for individual differences in disease severity, and analogous approaches have been used in previous FDG-PET subtyping studies in other neurodegenerative dementias [32, 34], as well as in MRI-based subtyping studies of neurodegeneration heterogeneity in AD (see, e.g. [3] for a recent review of this literature). We also note that the hypometabolic characteristics of the identified subtypes would not be consistent with the notion of merely reflecting variability in disease severity. As an example, the interpretation that the “limbic-predominant” subtype would reflect an earlier stage of the “typical” subtype cannot be easily reconciled with the observation of more severe medial temporal hypometabolism in the “limbic-predominant” subtype. A similar argument also applies to the comparison between the “typical” and “cortical-predominant” subtypes (also see Supplementary Figure 3 for a direct voxel-wise comparison of regional hypometabolic differences between the subtypes).

The contribution of disease stage to observed subtype phenotypes has been addressed in a recent MRI-based subtyping study by Young et al. [54], which proposed an analytical approach combining clustering with event-based modelling to assess subtypes and their respective stage progressions at the same time. However, this approach still relies on extrapolations from cross-sectional data. Future research on neurodegeneration subtypes in AD will benefit from longitudinal imaging assessments allowing to directly characterize disease progression within subtypes and to determine the possibility of conversion between them.

## Conclusion

In the current study, we used a systematic data-driven approach for characterizing differential neurodegeneration subtypes in AD as reflected by hypometabolism patterns on FDG-PET. The hypometabolic subtypes were associated with differential clinical and biomarker profiles, as well as with differences in clinical trajectories over time. These findings complement recent research efforts on characterizing distinct atrophy subtypes in AD using structural MRI data. Due to the reportedly higher sensitivity of FDG-PET for early neurodegenerative changes, the described hypometabolic subtypes may provide a sensitive tool for early detection and characterization of AD-related neurodegeneration variants at prodromal disease stages, which may have important implications for improving timely and differentiated prognosis in non-demented individuals with biomarker evidence of AD.

## Supplementary Information

**Additional file 1: Supplementary Table 1.** A/T/N profiles across AD and prodromal AD subtypes. **Supplementary Table 2.** Structures defined in the Harvard-Oxford atlas that were used to measure the composite cortical volume. **Supplementary Table 3.** Hazard ratios for progression of subtypes of patients with prodromal AD to dementia. **Supplementary Table 4.** Mixed effects regression models of longitudinal cognitive decline across subtypes in the prodromal AD group; “no hypometabolism” subtype as reference. **Supplementary Table 5.** Mixed effects regression models of longitudinal cognitive decline across subtypes in the prodromal AD group; “limbic-predominant” subtype as reference. **Supplementary Table 6.** Comparisons between AD subtypes with respect to the ratio of inferior to medial temporal metabolism assessed with FDG-PET. **Supplementary Table 7.** Correlations between the HV: CTV ratio and cognitive measures in the AD and in the prodromal AD groups. **Supplementary Table 8.** Missing values (at baseline) for demographic, clinical and biomarker characteristics in the AD dementia and prodromal AD groups. **Supplementary Figure 1.** Determination of optimal clustering cutoff by objective criteria. **Supplementary Figure 2.** Hierarchical clustering dendrogram and hypometabolic FDG-PET patterns of resulting AD subtypes at higher cluster solutions. **Supplementary Figure 3.** Comparisons of hypometabolic FDG-PET patterns of subtypes of patients with AD. **Supplementary Figure 4.** Hypometabolic FDG-PET patterns of subtypes of patients with prodromal AD with adjusted scale.

## Data Availability

Data analysed in this study were acquired from the Alzheimer’s Disease Neuroimaging Initiative (ADNI) database (http://adni.loni.usc.edu). ADNI data are shared in a de-identified form and without embargo subject to a review of a data use application by the ADNI Data Sharing and Publications Committee. For further information, please refer to the ADNI website (http://adni.loni.usc.edu/data-samples/access-data/).

## Abbreviations

AD: Alzheimer’s disease
ADNI: Alzheimer’s Disease Neuroimaging Initiative
ADNI-DIFF: Difference between ADNI-MEM and ADNI-EF
ADNI-EF: ADNI composite cognitive score for executive function
ADNI-MEM: ADNI composite cognitive score for memory function
APOE: Apolipoprotein E
Aβ: Beta-amyloid
CDR: Clinical Dementia Rating
CN: Cognitively normal
CSF: Cerebrospinal fluid
CTV: Cortical composite grey matter volume scaled to total intracranial volume
FDG: Fluorodeoxyglucose
FDR: False discovery rate
GM: Grey matter
HV: Hippocampal grey matter volume scaled to total intracranial volume
MCI: Mild cognitive impairment
MMSE: Mini-Mental State Examination
MRI: Magnetic resonance imaging
PET: Positron emission tomography
SUVR: Standard uptake value ratio
TIV: Total intracranial volume
WM: White matter
WMH: White matter hyperintensity volume

## Footnotes

1 The Elecsys β-Amyloid [1–42] CSF immunoassay in use is not a commercially available IVD assay. It is an assay that is currently under development and for investigational use only. The measuring range of assay is 200 (lower technical limit)—1700 pg/ml (upper technical limit). The performance of the assay beyond the upper technical limit has not been formally established. Therefore, the use of values above the upper technical limit, which are provided based on an extrapolation of the calibration curve, is restricted to exploratory research purposes and is excluded for clinical decision making or for the derivation of medical decision points.
