# Supplementary material for: Data-driven FDG-PET subtypes of Alzheimer’s disease-related neurodegeneration
Source: Alzheimers Res Ther. 2021 Feb 19;13:49. doi: 10.1186/s13195-021-00785-9 (PMC7896407; doi:10.1186/s13195-021-00785-9)
Supplement: Supplementary file 1 — Additional file 1: Supplementary Table 1. A/T/N profiles across AD and prodromal AD subtypes. Supplementary Table 2. Structures defined in the Harvard-Oxford atlas that were used to measure the composite cortical volume. Supplementary Table 3. Hazard ratios for progression of subtypes of patients with prodromal AD to dementia. Supplementary Table 4. Mixed effects regression models of longitudinal cognitive decline across subtypes in the prodromal AD group; “no hypometabolism” subtype as reference. Supplementary Table 5. Mixed effects regression models of longitudinal cognitive decline across subtypes in the prodromal AD group; “limbic-predominant” subtype as reference. Supplementary Table 6. Comparisons between AD subtypes with respect to the ratio of inferior to medial temporal metabolism assessed with FDG-PET. Supplementary Table 7. Correlations between the HV: CTV ratio and cognitive measures in the AD and in the prodromal AD groups. Supplementary Table 8. Missing values (at baseline) for demographic, clinical and biomarker characteristics in the AD dementia and prodromal AD groups. Supplementary Figure 1. Determination of optimal clustering cutoff by objective criteria. Supplementary Figure 2. Hierarchical clustering dendrogram and hypometabolic FDG-PET patterns of resulting AD subtypes at higher cluster solutions. Supplementary Figure 3. Comparisons of hypometabolic FDG-PET patterns of subtypes of patients with AD. Supplementary Figure 4. Hypometabolic FDG-PET patterns of subtypes of patients with prodromal AD with adjusted scale. [file 13195_2021_785_MOESM1_ESM.pdf]

Supplementary information

Data-driven FDG-PET subtypes of Alzheimer's disease-related neurodegeneration

Fedor Levin[1]; Daniel Ferreira[2]; Catharina Lange[3,4]; Martin Dyrba[1]; Eric Westman[2,5]; Ralph Buchert[6]; Stefan J. Teipel[1,7]; Michel J. Grothe[1,8*]; for the Alzheimer's Disease Neuroimaging Initiative[†]

[1]German Center for Neurodegenerative Diseases (DZNE), Rostock/Greifswald, Rostock, Germany

[2]Division of Clinical Geriatrics, Department of Neurobiology, Care Sciences and Society, Center for Alzheimer Research, Karolinska Institutet, Stockholm, Sweden

[3]Department of Nuclear Medicine, Charité – Universitätsmedizin Berlin, Corporate Member of Freie Universität Berlin, Humboldt-Universität zu Berlin, and Berlin Institute of Health, Berlin, Germany

[4]German Center for Neurodegenerative Diseases (DZNE), Dresden, Germany

[5]Department of Neuroimaging, Centre for Neuroimaging Sciences, Institute of Psychiatry, Psychology and Neuroscience, King's College London, London, UK.

[6]Department of Diagnostic and Interventional Radiology and Nuclear Medicine, University Medical Center Hamburg-Eppendorf, Hamburg, Germany

[7]Department of Psychosomatic Medicine, University of Rostock, Rostock, Germany

[8]Unidad de Trastornos del Movimiento, Servicio de Neurología y Neurofisiología Clínica, Instituto de Biomedicina de Sevilla, Hospital Universitario Virgen del Rocío/CSIC/Universidad de Sevilla, Seville, Spain.

[*]Corresponding Author:

Michel J. Grothe; Email: mgrothe@us.es

Supplementary table 1 A/T/N profiles across AD and prodromal AD subtypes.

	AD group, limbic-predominant subtype	AD group, typical subtype	AD group, cortical-predominant subtype	Prodromal AD group, no hypometabolism subtype	Prodromal AD group, limbic-predominant subtype	Prodromal AD group, typical subtype
Available data	71	80	11	52	105	45
**A+T-**	2 (3%)	10 (12.5%)	0 (0%)	5 (10%)	15 (14%)	5 (11%)
A+T-N-	2 (3%)	10 (12.5%)	0 (0%)	5 (10%)	14 (13%)	5 (11%)
A+T-N+	0 (0%)	0 (0%)	0 (0%)	0 (0%)	1 (1%)	0 (0%)
**A+T+**	69 (97%)	70 (87.5%)	11 (100%)	47 (90%)	90 (86%)	40 (89%)
A+T+N-	8 (11%)	7 (9%)	0 (0%)	11 (21%)	10 (10%)	7 (16%)
A+T+N+	61 (86%)	63 (79%)	11 (100%)	36 (69%)	80 (76%)	33 (73%)

Numbers represent participants in each subtype corresponding to specific A/T/N profiles. Percentages in parentheses represent share among participants in the given subtype who have available CSF data. The tau pathology biomarker "T" category was defined as a CSF p-tau level over 19.2 pg/ml; the neurodegeneration biomarker "N" category was defined as a CSF t-tau level over 242 pg/ml (1).

Supplementary table 2 Structures defined in the Harvard-Oxford atlas that were used to measure the composite cortical volume.

**Cortical region**	**Harvard-Oxford structure names**
Frontal	frontal pole
	middle frontal gyrus
	inferior frontal gyrus (pars triangularis)
	inferior frontal gyrus (pars opercularis)
Parietal	superior parietal lobule
	supramarginal gyrus (anterior division)
	supramarginal gyrus (posterior division)
	angular gyrus
Temporal	temporal pole
	superior temporal gyrus (anterior division)
	superior temporal gyrus (posterior division)
Occipital cortex	lateral occipital cortex (superior division)

Supplementary table 3 Hazard ratios for progression of subtypes of patients with prodromal AD to dementia.

	Model 1			Model 2		
Variable	Value	HR	z-statistic	Value	HR	z-statistic
Subtype	No hypometabolism subtype (reference)	1		No hypometabolism subtype	0.21***	-3.75
	Limbic-predominant subtype	4.82***	3.75	Limbic-predominant subtype (reference)	1	
	Typical subtype	5.99***	4.17	Typical subtype	1.24	0.75
Age		0.99	-0.66		0.99	-0.66
Gender		1.15	0.55		1.15	0.55
Education		1.06	1.25		1.06	1.25
Observations	200			200		
Number of events	71			71		

Hazard ratios are presented with z-statistics. * P < .05, ** P < .01, *** P < .001. Patients who did not progress to dementia within the observation period or did not have follow-up CDR scores were censored.

Supplementary table 4 Mixed effects regression models of longitudinal cognitive decline across subtypes in the prodromal AD group; “no hypometabolism” subtype as reference.

	Memory function composite score		Executive function composite score		Visuospatial function composite score		Language function composite score	
	Estimate	t-statistic	Estimate	t-statistic	Estimate	t-statistic	Estimate	t-statistic
Intercept	-0.177	-0.276	3.663***	4.758	0.736	1.373	1.009	1.554
Age	-0.002	-0.297	-0.05***	-5.426	-0.014*	-2.092	-0.024**	-3.011
Gender	0.218*	2.261	-0.043	-0.37	-0.003	-0.04	0.019	0.193
Education	0.054**	3.298	0.039*	1.985	0.022	1.575	0.08***	4.79
Follow-up time, months	-0.004*	-2.209	-0.005*	-2.068	-0.002	-0.581	-0.005*	-2.058
Limbic-predominant subtype	-0.478***	-3.91	-0.339*	-2.31	-0.027	-0.248	-0.388**	-3.137
Typical subtype	-0.759***	-5.696	-0.533**	-3.333	-0.237	-1.941	-0.324*	-2.402
Follow-up time × limbic-predominant subtype	-0.011***	-4.498	-0.009**	-2.903	-0.007*	-2.216	-0.009**	-2.762
Follow-up time × typical subtype	-0.012***	-3.875	-0.017***	-4.367	-0.015***	-3.532	-0.013**	-2.94

Unstandardized estimates are presented with t-statistics. * P < .05, ** P < .01, *** P < .001. For interactions between the follow-up time in months and subtype, the no hypometabolism subtype acts as a reference. Random intercepts and random slopes for participants are included to account for multiple measurements.

Supplementary table 5 Mixed effects regression models of longitudinal cognitive decline across subtypes in the prodromal AD group; “limbic-predominant” subtype as reference.

	Memory function composite score		Executive function composite score		Visuospatial function composite score		Language function composite score	
	Estimate	t-statistic	Estimate	t-statistic	Estimate	t-statistic	Estimate	t-statistic
Intercept	-0.655	-0.966	3.324***	4.065	0.709	1.245	0.621	0.901
Age	-0.002	-0.297	-0.05***	-5.426	-0.014*	-2.092	-0.024**	-3.011
Gender	0.218*	2.261	-0.043	-0.37	-0.003	-0.04	0.019	0.193
Education	0.054**	3.298	0.039*	1.985	0.022	1.575	0.08***	4.79
Follow-up time, months	-0.015***	-10.047	-0.014***	-7.2	-0.009***	-4.209	-0.015***	-7.059
Typical subtype	-0.281*	-2.315	-0.194	-1.33	-0.21	-1.882	0.064	0.523
No hypometabolism subtype	0.478***	3.91	0.339*	2.31	0.027	0.248	0.388**	3.137
Follow-up time × typical subtype	-0.001	-0.331	-0.008*	-2.25	-0.008	-1.931	-0.003	-0.808
Follow-up time × no hypometabolism subtype	0.011***	4.498	0.009**	2.903	0.007*	2.216	0.009**	2.762

Unstandardized estimates are presented with t-statistics. * P < .05, ** P < .01, *** P < .001. For interactions between the follow-up time in months and subtype, the limbic-predominant subtype acts as a reference. Random intercepts and random slopes for participants are included to account for multiple measurements

Supplementary table 6 Comparisons between AD subtypes with respect to the ratio of inferior to medial temporal metabolism assessed with FDG-PET.

	AD group, limbic-predominant (S1)	AD group, typical (S2)	AD group, cortical-predominant (S3)	P-value, Global comparison (S1, S2 and S3)	Pair-wise comparisons		
					S1 vs S2	S1 vs S3	S2 vs S3
Inferior/medial temporal metabolism	1.30 (0.13)	1.21 (0.10)	1.11 (0.07)	< 0.001	< 0.001	< 0.001	0.009

The ratio of inferior to medial temporal metabolism was calculated as the average signal in the inferior temporal gyrus (anterior and posterior parts) divided by the signal in the combined hippocampus and amygdala regions as defined in the Harvard-Oxford atlas.

Values are presented as means with standard deviation in parentheses. Subtypes were compared with post-hoc pairwise t-tests with FDR correction. S1 = limbic-predominant subtype; S2 = typical subtype; S3 = cortical-predominant subtype.

Supplementary table 7 Correlations between the HV:CTV ratio and cognitive measures in the AD and in the prodromal AD groups.

	AD group	Prodromal AD group
	HV:CTV ratio	HV:CTV ratio
ADNI-MEM	-0.140	0.125
ADNI-EF	-0.373***	-0.120
ADNI-DIFF	0.339***	0.244***
ADNI-VS	-0.276***	0.095
ADNI-Lan	-0.228**	-0.007
MMSE	-0.046	0.057

* P < .05, ** P < .01, *** P < .001

HV:CTV ratio: ratio of hippocampal grey matter volume to cortical composite grey matter volume.

Supplementary table 8 Missing values (at baseline) for demographic, clinical and biomarker characteristics in the AD dementia and prodromal AD groups.

	CN group (n = 179)	AD group, limbic-predominant subtype (n = 79)	AD group, typical subtype (n = 86)	AD group, cortical-predominant subtype (n = 12)	Prodromal AD group, no hypometabolism subtype (n = 57)	Prodromal AD group, limbic-predominant subtype (n = 108)	Prodromal AD group, typical subtype (n = 49)
	n missing	n missing	n missing	n missing	n missing	n missing	n missing
**Demographics**							
Age	0	0	0	0	0	0	0
Sex	0	0	0	0	0	0	0
Education	0	0	0	0	0	0	0
**ApoE genotype**							
ApoE-ε4	3	2	2	0	0	1	1
**Cognition**							
MMSE	0	0	0	0	0	0	0
ADNI-MEM	0	0	0	0	0	0	0
ADNI-EF	0	0	0	0	0	0	0
ADNI-DIFF	0	0	0	0	0	0	0
ADNI-VS	0	0	0	0	0	0	0
ADNI-Lan	0	0	0	0	0	0	0
**Biomarkers**							
AV45-PET SUVR	0	22	25	1	0	0	0
CSF Aβ	21	8	6	1	5	3	4
CSF t-tau	21	8	6	1	5	3	4
CSF p-tau	22	8	6	1	5	3	4
HV, adjusted to the	0	1	2	0	0	0	0

TIV							
CTV, adjusted to the TIV	0	1	2	0	0	0	0
HV:CTV ratio	0	1	2	0	0	0	0
WMH	3	7	5	1	1	0	1

Supplementary figure 1. Determination of optimal clustering cutoff by objective criteria.

Two different objective criteria were used for determining the optimal number of clusters in the hierarchical clustering analysis. Respective criteria are plotted as y-values, whereas x-values represent numbers of clusters.

A - Davies-Bouldin criterion. Lower values indicate better clustering solutions. B - Silhouette criterion. Higher values indicate better clustering solutions.

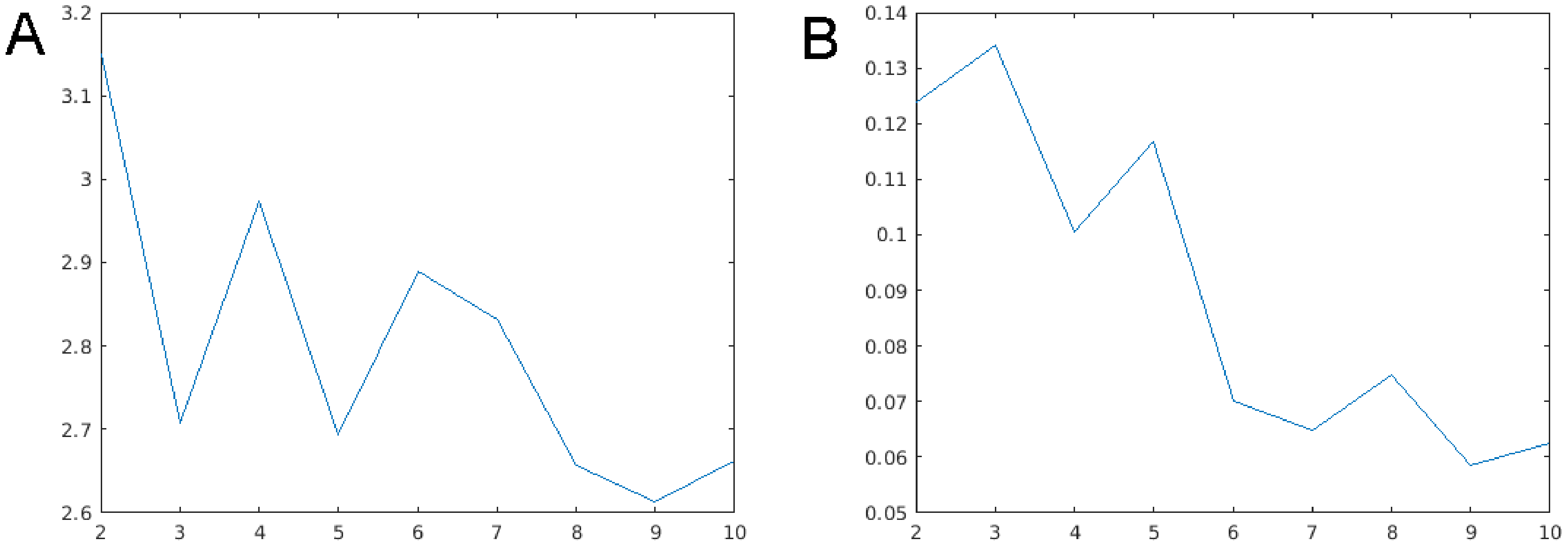

Supplementary figure 2. Hierarchical clustering dendrogram and hypometabolic FDG-PET patterns of resulting AD subtypes at higher cluster solutions.

A Five-cluster hierarchical clustering solution. Compared to the three-cluster solution, here the "typical" subtype is split into three subdivisions, which show a similar temporo-parietal pattern of hypometabolism, but with different degrees of lateralization.

B Six-cluster hierarchical clustering solution as produced by splitting the limbic-predominant cluster into two separate clusters suggested by the dendrogram structure (split depicted by the dashed line on the dendrogram). This step separates a patient subgroup with only minimal hypometabolism.

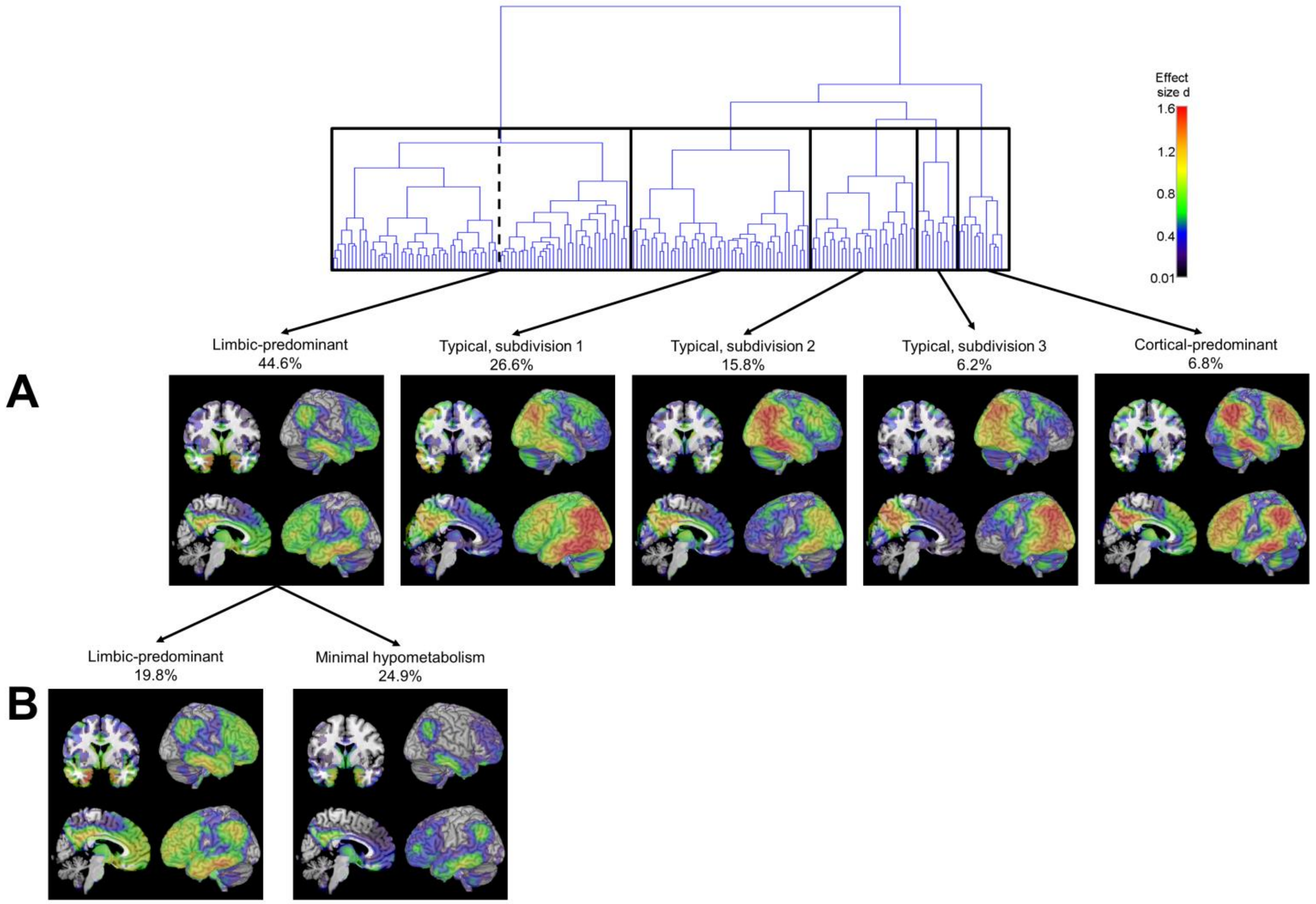

Supplementary figure 3. Comparisons of hypometabolic FDG-PET patterns of subtypes of patients with AD

Voxel-wise hypometabolic patterns of the three AD subtypes were compared to each other. FDG-PET scans were scaled to the pons reference signal prior to analysis, and age, gender, years of education and MMSE were used as covariates. Statistical parametric maps of the group differences were converted into Cohen’s d effect size maps to allow for a better comparison of the patterns across the unevenly sized subgroups. Please note, that in this figure the scales for effect size maps range from -1.1 to -0.01, and from 0.01 to 1.1.

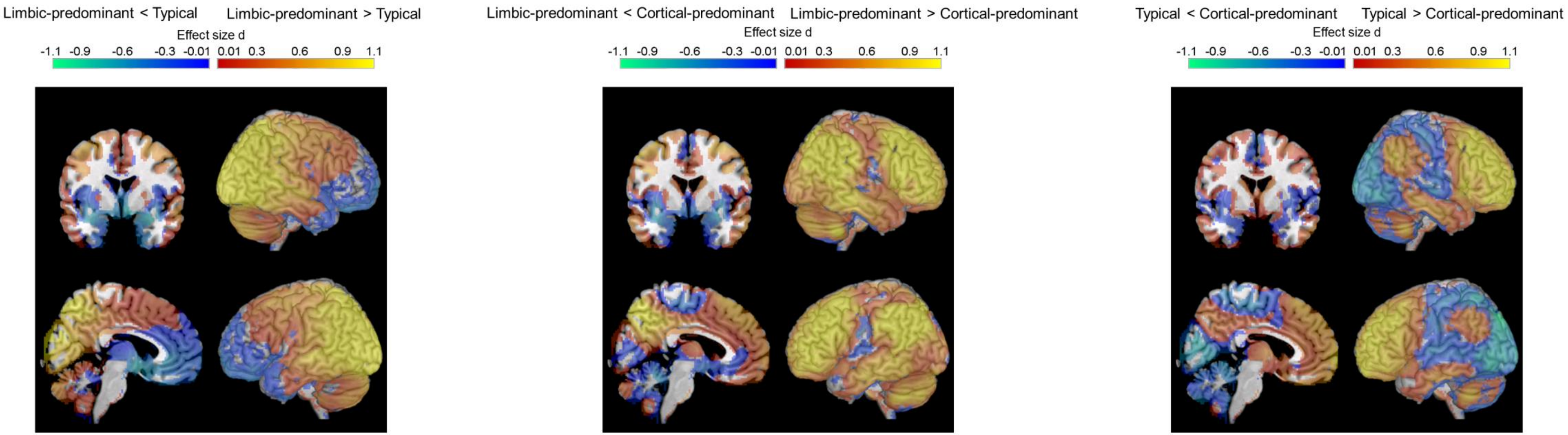

Supplementary figure 4. Hypometabolic FDG-PET patterns of subtypes of patients with prodromal AD with adjusted scale

Voxel-wise hypometabolic patterns of the four prodromal AD subtypes as compared to the healthy control group. FDG-PET scans were scaled to the pons reference signal prior to analysis, and age, gender, and years of education were used as covariates. Statistical parametric maps of the group differences were converted into Cohen's d effect size maps to allow for a better comparison of the patterns across the unevenly sized subgroups. Please note that in this Figure the patterns are displayed at the same Cohen's d scale as the hypometabolic patterns of the AD dementia subtypes illustrated in Fig. 1 of the main text (i.e. from 0.01 to 1.6).

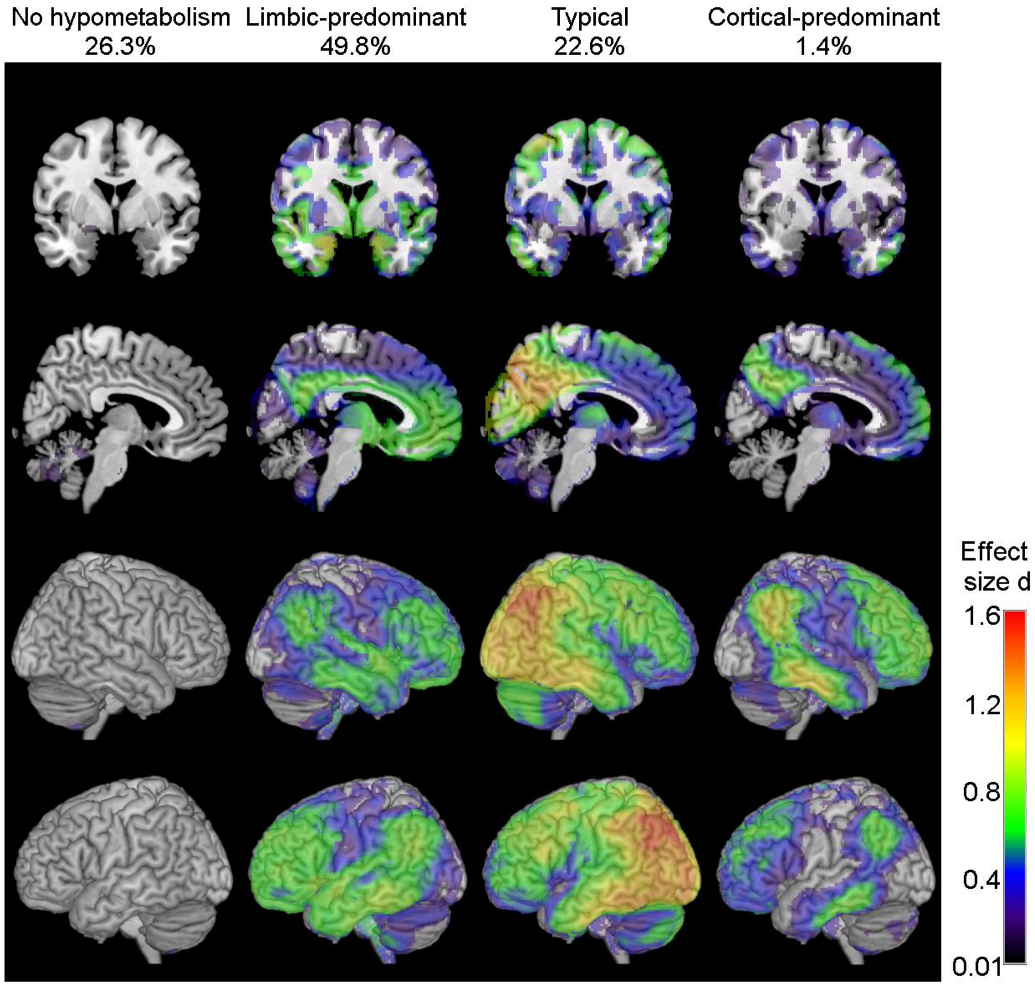

References

1. Salvado G, Molinuevo JL, Brugulat-Serrat A, Falcon C, Grau-Rivera O, Suarez-Calvet M, et al. Centiloid cut-off values for optimal agreement between PET and CSF core AD biomarkers. Alzheimers Res Ther. 2019;11(1):27.
